# Available Assistive Technology Outcome Measures: Systematic Review

**DOI:** 10.2196/51124

**Published:** 2023-11-15

**Authors:** Francesca Borgnis, Lorenzo Desideri, Rosa Maria Converti, Claudia Salatino

**Affiliations:** 1 IRCCS Fondazione Don Carlo Gnocchi ONLUS Milan Italy; 2 AIAS Bologna onlus Bologna Italy

**Keywords:** assistive technology, AT, AT assessment, AT outcome measures, rehabilitation

## Abstract

**Background:**

The World Health Organization claimed that measuring outcomes is necessary to understand the benefits of assistive technology (AT) and create evidence-based policies and systems to ensure universal access to it. In clinical practice, there is an increasing need for standardized methods to track AT interventions using outcome assessments.

**Objective:**

This review provides an overview of the available outcome measures that can be used at the follow-up stage of any AT intervention and integrated into daily clinical or service practice.

**Methods:**

We systematically searched for original manuscripts regarding available and used AT outcome measures by searching for titles and abstracts in the PubMed, Scopus, and Web of Science databases up to March 2023.

**Results:**

We analyzed 955 articles, of which 50 (5.2%) were included in the review. Within these, 53 instruments have been mentioned and used to provide an AT outcome assessment. The most widely used tool is the Quebec User Evaluation of Satisfaction with Assistive Technology, followed by the Psychosocial Impact of Assistive Technology Scale. Moreover, the identified measures addressed 8 AT outcome domains: functional efficacy, satisfaction, psychosocial impact, caregiver burden, quality of life, participation, confidence, and usability. The AT category *Assistive products for activities and participation relating to personal mobility and transportation* was the most involved in the reviewed articles.

**Conclusions:**

Among the 53 cited instruments, only 17 (32%) scales were designed to evaluate specifically assistive devices. Moreover, 64% (34/53) of the instruments were only mentioned once to denote poor uniformity and concordance in the instruments to be used, limiting the possibility of comparing the results of studies. This work could represent a good guide for promoting the use of validated AT outcome measures in clinical practice that can be helpful to AT assessment teams in their everyday activities and the improvement of clinical practice.

## Introduction

### Background

The World Health Organization (WHO) has defined assistive technologies (ATs) as the fourth pillar of global health, along with drugs, vaccines, and medical devices [1]. Specifically, assistive products (APs) must be considered necessary to maintain or improve a person’s functioning. Indeed, it is well known that AT has the potential to sustain people living with limitations owing to age, disease, or disability in maintaining or improving their functioning and independence with a positive impact on mobility, social interaction, and the quality of life of patients and those around them [1,2]. In 2021, over 1 billion people globally needed ≥1 AT, a number that is expected to double by 2050 [1]. Despite the overt need for ATs, the evidence on the real ability of APs to reduce the impact of disease or disability in the user’s life is still poor; reliable data on the need for AT and its outcomes are limited [3]. The AT outcome has been indicated by the WHO as among the 5 top priorities in AT research [4]. However, only few countries in the WHO European Region have comprehensive monitoring mechanisms to evaluate the AT need and the impact on disease in the patient’s life [5]. The need for evidence-based strategies in this field is also mentioned in the global report on AT published by the United Nations International Children’s Emergency Fund and WHO in 2022 “Measuring outcomes and impact is necessary to understand the benefits of AT and create evidence-based policies and systems to ensure universal access to it” [1]. It is well known that the quality of the assessment has a wide impact on the user’s experience with an AT, in addition to the quality of their interactions with the AT [6]. Stemming from the seminal work of Fuhrer et al [7], who defined the outcome in the context of AT provision as a “systematic investigation aimed at identifying the changes that are produced by AT in the lives of users and their environment,” research more recently has started to emphasize the distinction between the outcomes and impact of AT. Specifically, the Global Alliance of Assistive Technology Organizations refers to outcomes as finite and measurable changes that occur in response to an intervention such as AT [8]. AT outcome assessment usually focuses on the short-term effect of an AT intervention (ie, provision of AT and its implementation in the user’s life and context). Evidence from outcome research shows that AT enables people of all ages with any type of disability to overcome their functional difficulties, supporting them in achieving an important life [1]. On the other side, impact refers to broader changes that occur within the community as a result of outcomes [8]. As such, the impact is generally considered rather challenging to define and measure compared with the outcomes. This review focuses on assessing the outcome of providing an AT. In clinical practice, there is a growing need for standardized methods to track individual AT interventions by means of outcome assessment [9]. The use of internationally validated AT outcome measures can be helpful to AT assessment teams in their everyday activities and informs the improvement of clinical practice. Furthermore, different stakeholders are involved in the AT service delivery process (service manager, clinic director, different therapists, social workers, psychologists, rehabilitation engineers, funding agencies, AT users, and caregivers), and they see AT outcomes from different perspectives and might be interested in different aspects of AT outcomes [10].

As recently observed, measuring AT service delivery outcomes may be instrumental for any AT system to document evidence at individual, service, and system levels [2]. At the individual level, it allows AT and professionals to monitor their interventions and routinely perform corrective actions when necessary. At the service level, it facilitates the assessment and monitoring of the overall functioning of a specific service delivery process over time, as well as assuring the continuous involvement of all stakeholders. At the system level, outcome assessment allows the identification of differences in service delivery practices, processes, programs, and policies, thus providing policy makers with a reliable evidence base upon which the consequences (eg, factors) associated with these differences can be identified and addressed.

In the AT service delivery process, the most appropriate time to implement outcome measurement is at the follow-up, after a reasonable time of use of the APs by the users in their real living environment [11] to be able, if necessary, to implement corrective or improvement actions. Indeed, over the years, several studies have focused on the high rate of abandonment of received ATs owing to changes in health conditions [12] or failure of ATs to meet patients’ or closest relatives’ needs and expectations [6]. An AT solution brings about a disruption in the “system composed of the person, his or her environment and occupation” [13]. The system needs time to absorb the disruption and evolve toward a new balanced situation; the outcome is positive when this new situation is perceived by the person and by his or her primary network as beneficial to their life [14]. A variety of actors and factors are involved in this system, some of them being predictable and others unpredictable; thus, the actual outcomes can be detected only when the disruption transient has expired: outcome measurement should be carried out not *in the clinic* but *in the real environment* and not *here and now* but *there and tomorrow* [15].

A recent scoping review has been conducted to chart the landscape and development of AT evaluation tools across disparate fields [16]. In light of the increasing need for standardized methods to track individual AT interventions, we focused on clinical practice, particularly AT outcome measures within the rehabilitation path. In this context, a systematic review of the literature published in the last 2 decades has been undertaken to provide an overview of available outcome measures that can be used at the follow-up stage of any AT intervention and integrated into the daily clinical or service practice.

### Objectives

This systematic review aims to answer the following research questions:

1. What AT intervention outcomes do the available instruments allow for evaluating?
2. To which categories of AT have the available measures been applied?

This review might be considered relevant for AT practitioners and researchers as, to our knowledge, no published systematic investigation of the extant literature has been recently performed to provide a comprehensive examination of available and used AT outcome measures.

## Methods

This systematic review was conducted using the PRISMA (Preferred Reporting Items for Systematic Reviews and Meta-Analyses) guidelines and a flow diagram [17]. The protocol for this review was registered in the PROSPERO (registration number CRD 42022338395).

### Eligibility Criteria

**Figure 1:**
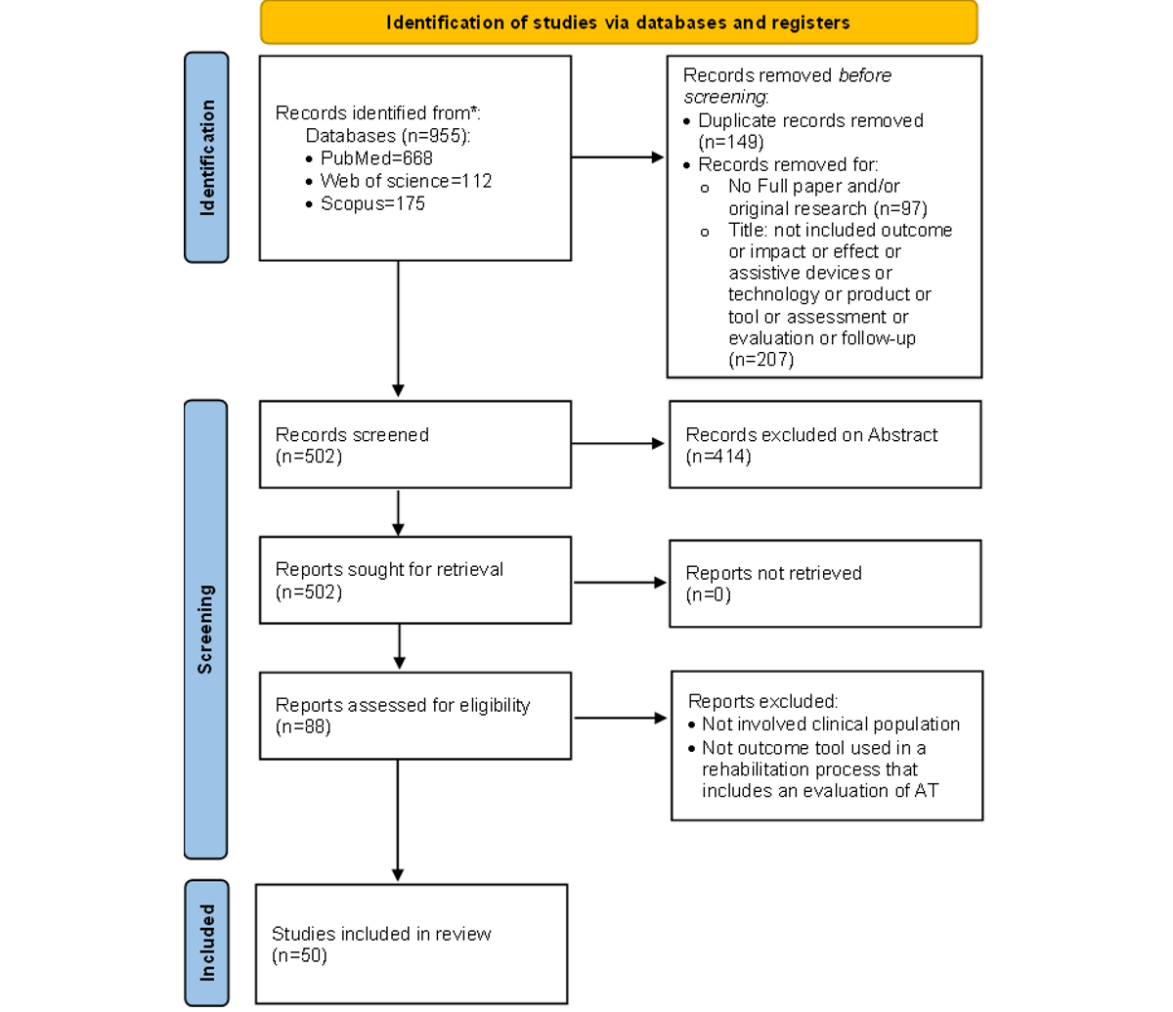
Overall procedure of the study and the number of selected articles. AT: assistive technology.

Studies were included if they fulfilled the following inclusion criteria: (1) original studies, (2) involving AT outcome or impact assessment, (3) published since 2002, and (4) published in the English language. As defined by the WHO, AT is an umbrella term that covers the systems and services related to the delivery of APs and services, including hearing aids, wheelchairs, communication aids, spectacles, prostheses, pill organizers, and memory aids [1]. For a comprehensive review, we included all the APs. The exclusion criteria were non–full-text papers (ie, books, chapters of the books, qualitative studies, letters, comments, dissemination, and published abstracts without text), published in a non-English language, and involved a sample aged ≤18 years. The overall procedure of the study and the number of selected articles are shown in Figure 1. After searching the electronic databases, 955 articles were collected. After removing duplicates and outliers (n=149), the remaining articles were used in the next phase. In the first screening phase, 304 articles were excluded based on the titles (n=207) and exclusion criteria (n=97). A second screening phase was conducted in which 414 articles were excluded by the abstract. A full-text review was conducted (n=88), and 38 (43%) papers were excluded based on the inclusion criteria. Finally, 50 articles were selected for the qualitative synthesis.

### Information Sources for the Study Selection

Articles were searched in the PubMed, Web of Science, and Scopus electronic databases from 2002 to March 2023 (last 20 y). These databases appear adequate to cover the broad spectrum of topics in the target area [18]. Bibliographies identified articles, and a manual search of relevant journals for additional references was conducted. We used the search query string ((assistive technology intervention) AND ((outcome measure*) OR (impact measure*))) AND (adult*) (the asterisk indicates that the search term was not limited to that word).

### Selection and Data Collection Process

Eligibility screening was blindly and independently conducted by 2 researchers on the Rayyan platform [19]. Interreviewer disagreements were resolved by discussion to reach a consensus or by a third reviewer when the agreement was not reached. After selecting the studies, data collection focused on the demographic and clinical characteristics of patients, AT (including the category of assistive devices [ADs]), and AT outcome measures.

## Results

### Overview

In the last decade (2013-2022), the studies on outcome measures assessment have almost tripled compared with those in the previous decade (2002-2012). Indeed, 14 studies were conducted between 2002 and 2012 and 36 studies were conducted between 2013 and 2022. Moreover, in the last 2 years, there has been a substantial increase in studies in this area (Multimedia Appendix 1). The 50 papers included in this review can offer an overview of AT outcome or impact measures to try to answer our research question: “What AT outcome measures are currently available and used?”

To date, 53 instruments have been mentioned and used to provide an AT outcome measure assessment (Multimedia Appendix 2 [20-66]). In addition, 3 ad hoc questionnaires and an ad hoc interview were used.

The most widely used tool was the Quebec User Evaluation of Satisfaction with Assistive Technology (QUEST 2.0; 12/50, 24%), followed by the Psychosocial Impact of Assistive Technology Scale (9/50, 18%), Wheelchair Skills Test Questionnaire (WST-Q; 8/50, 16%), Wheelchair Use Confidence Scale for power wheelchair users (WheelCon-P; 6/50, 12%), and Caregiver Assistive Technology Outcome Measure (CATOM; 5/50, 10%). Another 3 tools have been mentioned in 4 papers, that is, Wheelchair Outcome Measure (WhOM), Canadian Occupational Performance Measure (COPM), and Spinal Cord Injury (SCI)–Functional Index (FI) or AT. Five tools were described in 3 papers, followed by 6 instruments used 2 times. The remaining 34 instruments were mentioned once.

The instruments differ with regard to the intervention outcomes domains considered and the category or type of AT to which they were applied. To create a reasoned and organized analysis of the available outcome measures for AT assessment, we have structured the *Results* section into paragraphs, starting with 2 questions.

### What Intervention Outcomes Do the Available Instruments Allow for Evaluating?

The review of articles showed that 8 domains were evaluated by the available and used instruments. The most evaluated intervention outcome is “Functional Efficacy,” investigated in 72% (36/50) of the papers. The second most analyzed domain is satisfaction (28/50, 56%), followed by psychosocial impact (12/50, 24%), caregiver burden (6/50, 12%), quality of life (9/50, 18%), participation (8/50, 16%), confidence (6/50, 12%), and usability (3/50, 6%).

#### Functional Efficacy

##### Overview

AT can contribute to the achievement of enhanced functioning from an International Classification of Functioning perspective. Efficacy can be assessed by evaluating a variety of variables. Of the 50 reviewed studies, 36 (72%) have investigated this domain. Overall, 26 scales were used to evaluate the functional efficacy. Of these, 6 (23%) instruments were specifically designed and developed for evaluating APs. The most commonly used questionnaire is the WST-Q, which appears to be an outcome measure in 9 studies.

##### Wheelchair Skills Test Questionnaire

The WST-Q is a standardized evaluation method developed and used to self-evaluate manual or powered wheelchair skills and safety [67]. In addition to assessing capacity (as in Wheelchair Skills Test; WST), the WST-Q assesses confidence and performance (what wheelchair users do and how they do it). In the reviewed articles, it was administered to patients and their informal caregivers [20,21]. With regard to patients, the studies showed that it is an effective way to evaluate variation in 32 power mobility skills (eg, the capability of putting on brakes, propelling a straight distance, and performing a reaching task from their wheelchair) in all power mobility AP users [22]. For example, studies used the WST-Q to evaluate the functional efficacy in patients with autosomal recessive spastic ataxia [23], multiple sclerosis, and SCI [24].

Furthermore, 22% (2/9) of the studies also reported the *WST* as an instrument to confirm the WST-Q results [25,26]. However, a study showed that the WST can be considered a reliable, valid, and useful tool for evaluating safety and performance domains in several clinical populations who need manual wheelchairs, specifically amputations, stroke, musculoskeletal disorders, SCI, and neuromuscular disorders [27].

##### SCI-FI or AT Tool

The SCI-FI or AT banks allow for assessing an individual’s ability to use new APs and how the ability to execute day-to-day functional activities (with AT) changes over time [28]. It focuses on the ability to perform activities, as usual, using APs, specifically basic mobility (eg, changing or maintaining body positions and transfers), self-care (eg, eating and dressing), fine motor (eg, manipulating and moving objects), ambulation, and wheelchair mobility. Four studies involving large samples (from 269 patients to 1237 patients) of patients with SCI used SCI-FI or AT to assess mobility ADs outcome such as wheelchairs [29,30]. Studies converge in supporting that SCI-FI or AT is an optimal solution to evaluate functioning with AT in 4 domains: basic mobility, self-care, fine motor function, and ambulation [31].

##### Life-Space Assessment

Overall, 3 studies used this self-report measure to quantify “how far and how often they have mobilized with or without assistance” during the last 4 weeks. In detail, this tool investigates power mobility in 5 “Life-Space Levels” (bedroom or sleeping area, external area of the residence, neighborhood, and inside and outside the city). The studies involved patients who required several mobility APs, including mobility service dogs and manual and powered wheelchairs. The reviewed articles showed that this tool can be administered to patients with diseases of the nervous system and sense organs, such as autosomal recessive spastic ataxia [23] or multiple sclerosis [24] and traumatic damage to the spinal cord [26].

Three questionnaires were cited in 2 studies for each to evaluate the functional efficacy of APs in terms of “functional independence,” “functional mobility,” and “functional communication.”

##### Functional Independence Measure

The Functional Independence Measure (FIM) assesses the degree of assistance required by patients to perform motor (eg, eating, grooming, bathing, and dressing) and cognitive (eg, comprehension and expression) activities of daily living [68]. In the reviewed papers, the tool was used to assess the patient’s level of disability as well as a change in patient status consequent to the AT intervention. The FIM has been used to evaluate changes in patients with several motor disabilities, including deficits because of traumatic SCI [29]. The reviewed studies showed that the FIM can be used to evaluate the functional efficacy of 2 main categories of ADs: products for controlling, carrying, moving, and handling objects and devices (ie, smart environment) and products for mobility (ie, wheelchairs) [29,32].

In their study, Tyner et al [29] adopted a modified version of FIM, the *Self-Report Functional Measure*, an alternative brief self-report instrument developed to measure 13 different motor functions affecting basic activities of daily living in wheelchair users with SCI.

##### Functional Mobility Assessment

The Functional Mobility Assessment (FMA) was adapted from the *Functional Everyday with a Wheelchair* (FEW) questionnaire, which included all items relevant to individuals who use mobility devices, that is, wheelchairs, scooters, canes, crutches, or walkers that allow them to perform functional tasks independently, safely, and efficiently as possible [33]. It measures the perceived functional independence of individuals in several tasks, such as “carrying out daily routine” or “personal care tasks.” The 2 reviewed studies applied the FMA as a functional performance efficacy measure applied to mobility APs (eg, wheelchairs, scooters, canes, crutches, and walkers).

FEW is a brief, structured self-report outcome measurement questionnaire used to evaluate the functioning in wheelchair users [69]. Only 1 reviewed study has used this tool to evaluate the performance in the actually lived environment of users who need manual and power mobility devices [34].

##### Functional Assessment of Communication Skills for Adults

The Functional Assessment of Communication Skills for Adults (FACS-A) is a 43-item scale that measures functional communication in daily living activities (eg, understanding television and radio, responding in an emergency, and using a calendar) [70]. In detail, it focuses on 4 domains: social communication; communication of basic needs; reading, writing, and number concepts; and daily planning. In their study, Vincent et al [35] used only items of the first 2 domains that might be affected by using APs with hearing persons. Specifically, FACS-A was used to evaluate changes in communication in hearing persons using “Le communicateur Oralys on pocket PC,” a software that translates sign language into oral French. In another study, FACS-A was adopted to evaluate functional communication in patients with cerebral palsy using augmentative and alternative communication [36].

##### Further Functional Efficacy Scales

The other 17 instruments were cited by only 1 article each. Among them, only 2 instruments have been developed for evaluating the AT intervention: the (1) *Wheelchair User’s Shoulder Pain Index (WUSPI)* [71] and *Individually Prioritised Problem Assessment (IPPA)* [72,73]. *WUSPI* was used in a study involving adults with traumatic SCI, which showed the validity of WUSPI as a self-report measure to evaluate the effect of mobility service dogs on shoulder pain in wheelchair users during functional activities (eg, transfers, wheelchair mobility, self-care, and general activities) [26].

The other tools have not been specifically designed and developed for AT interventions. They allow for evaluating several aspects of functional efficacy and have been used according to the prescribed APs. For example, activities that can be performed using ADs can be broken down into several tasks, and assessing *difficulty in carrying them out* can be an efficacy indicator. In the literature, 3 instruments evaluating this aspect have been found: the (2) *IPPA,* the (3) *COPM* [74], and (4) *Assessment of Life Habits (LIFE-H)* [75]. IPPA is a validated tool that evaluates the perceived effectiveness of an AT intervention. Mortenson et al [37] showed that IPPA can be successfully used with different AT categories and clinical populations. Specifically, in the reviewed article, IPPA was used to evaluate the ability of AT interventions involving APs for personal mobility and domestic activities to improve older AT users’ activity performance. COPM is a client-centered outcome measure for patients to identify and prioritize everyday issues that restrict their participation in everyday life. LIFE-H is a self-report measure to capture the self-rated level of accomplishment for everyday activities of people with disabilities, collecting information on all life habits that people carry out in their environments (home, workplace or school, and neighborhood). One study involved in this review showed that LIFE-H can be used in patients with autosomal recessive spastic ataxia and users of manual and powered wheelchairs [38]. Overall, 6 instruments described in 2 articles were available and were used to evaluate functional efficacy in *cognitive abilities*. A study has focused on changes in many cognitive domains (eg, orientation, memory, attention, and language) of older adults with or without dementia that uses an electric calendar to compensate for time orientation and memory [39]. The authors investigated changes in cognitive functioning using 2 screening tests: (5) *Mini-Mental Examination State* [76] and (6) *Neurobehavioral Cognitive Status Examination* [77]. Moreover, they investigated whether the chosen AP could impact the behaviors in daily living, measured by the short version of the (7) *Dementia Behavior Disturbance Scale*, a validated tool for behavioral and psychological symptoms in people with dementia [78]. Other studies have focused on specific cognitive domains, particularly language. The (8) *Communicative Effectiveness Index modified* was used by Londral et al [40] to measure the efficiency of assistive communication devices in supporting speech dysfunction in patients with amyotrophic lateral sclerosis. Another study evaluated the impact of assistive communication products in the language domain, focusing on receptive language skills, reading comprehension, and functional communication [36]. In detail, they evaluated patients with cerebral palsy using the (9) *Gray Silent Reading Test* for reading comprehension ability [79]; the (10) *Test of Auditory Comprehension of Language, revised* for comprehension of semantics, morphology, and syntax; and the (11) *Peabody Picture Vocabulary Test*, revised [80] for receptive (hearing) vocabulary.

Overall, 4 studies have focused on *functional autonomy/independence* related to AT interventions. First, Mortenson et al [41] assessed mobility independence and performance in activities of daily living in patients who needed mobility APs using 3 subscales of the (12) *Functional Autonomy Measurement System* [81]. The (13) *Barthel Index for Activities of Daily Living* was another tool used to measure functional independence in activities of daily living [82]. In the reviewed study, this instrument allowed for investigating changes in functional independence in performing everyday activities (eg, bathing, grooming, dressing, chair transfer, and mobility) in patients with autosomal recessive spastic ataxia who use manual and powered wheelchairs [38]. Similarly, the (14) *Spinal Cord Independence Measure III* has been used to evaluate any changes in performing activities of daily living and mobility in patients with SCI using manual and powered wheelchairs [42]. Finally, the (15) *Occupational Therapy Functional Assessment Compilation (OTFAC)* tool [43], which was initially designed as a comprehensive functional assessment tool for occupational therapists, can also be used to isolate the impact of an AT intervention on a person’s functional performance. The reviewed article showed the feasibility of OTFAC as a tool for evaluating changes in functional autonomy in several clinical conditions using mobility APs such as wheelchairs and seating systems [43].

Finally, 2 studies have investigated functional changes in *basic and instrumental daily life activities* in 2 clinical populations (ie, dementia and amyotrophic lateral sclerosis). First, the (16) *Bristol Activity of Daily Living Scale* (BADLS) [83], which focuses on independence in performing 20 activities, was developed specifically to be used with patients affected by dementia. The reviewed study used the BADLS to evaluate whether AT interventions extend the time that people with dementia can continue to live independently at home [44]. In addition, the (17) *Amyotrophic Lateral Sclerosis Functional Rating Scale* is another valid scale to evaluate the patients’ functioning in performing activities of daily living, making use of the prescribed assistive communication devices [40].

#### Satisfaction

##### Overview

In the standard “ISO 9241-11:2018 Ergonomics of human-system interaction—Part 11: Usability,” the definition of satisfaction can also be found: “extent to which the user’s physical, cognitive and emotional responses that result from the use of a system, product or service meet the user’s needs and expectations.”

The high abandonment rates of the AT are associated with the device’s poor performance, which does not meet the environmental needs and does not consider the user’s opinion [22]. The effective use of evidence-based strategies is particularly necessary because the level of availability of AT, especially wheelchairs, is low and can be abandoned if inappropriate for the user. Wheelchair users often have problems such as, for example, discomfort and poor posture. Therefore, it is essential to investigate user satisfaction by involving users in a collaborative target identification process [45]. However, there are only a few wheelchair-specific measures. Overall, 18 articles focused on evaluating patients’ satisfaction with using the prescribed AT devices. A total of 5 instruments were used.

##### QUEST 2.0 Tool

QUEST 2.0 allows for evaluating the individuals’ satisfaction with the AT equipment they are using [84]. The reviewed articles showed that QUEST 2.0 is the most used questionnaire in AT outcome assessment. Papers appear heterogeneous in terms of AT categories and clinical populations. First, QUEST 2.0 can be successfully administered directly to patients to obtain the opinion of those using the APs. In more detail, QUEST 2.0 has been administered to many clinical conditions, including SCI [26], neuromuscular or musculoskeletal disorders (eg, rheumatoid arthritis) [46], and diseases of the nervous system and sense organs (eg, low vision) [35,47]. With regard to the evaluated ADs, QUEST 2.0 was used to evaluate user satisfaction in using 4 different categories of ADs: mobility [26], communication and information management [47,48], domestic activities [46,49], and controlling devices [32].

##### AT Device Predisposition Assessment

Assistive Technology Device Predisposition Assessment (ATD-PA) is one of the most well-known instruments for evaluating the overall user experience with AT. Only 1 study used the questionnaire ATD-PA (device form) to evaluate consumers’ subjective satisfaction [50]. This study did not provide detailed information on the ATs or populations that may use it. Therefore, this tool is a stable, valid, and reliable instrument for evaluating products and services, and is potentially usable for all patients who need ATs.

##### LIFE-H Tool

The LIFE-H is a self-report measure of the perceived level of accomplishment for everyday activities of people with disabilities that includes a scale evaluating the individual’s satisfaction regarding the accomplishment of life habits [75]. Moreover, 3 of the reviewed articles showed that this tool can be administered to patients with disabilities affected by diseases of the nervous system and sense organs, such as autosomal recessive spastic ataxia [38]. The studies applied the LIFE-H to 3 main categories of ATs, specifically mobility [37,38], communication and information management [35], and domestic activities [37].

##### WhOM Tool

WhOM is the second most commonly used patient-centered measure for evaluating satisfaction with the performance of self-identified activities. Unlike QUEST 2.0 and ATD-PA, it is a specific instrument for wheelchair and seating systems intervention [85]. The reviewed articles showed that this instrument has been used only in patients with 2 broad clinical categories: suspected or confirmed neurodegenerative conditions, such as autosomal recessive spastic ataxia [38], and traumatic damage to the spinal cord [51,52].

##### Wheelchair Satisfaction Questionnaire

The Wheelchair Satisfaction Questionnaire (WSQ) is designed to provide data on wheelchair users’ satisfaction with their AP at a given moment [53]. The only study mentioning this questionnaire supported its potential to give wheelchair users a quantifiable voice on wheelchair function [53]. It is well known that providing data from wheelchair users to wheelchair manufacturers and providers leads to a better design and provision.

#### Psychosocial Impact

The psychosocial impact can be seen as the impact on AT users’ psychosocial well-being, including subjective perceptions of the changes that occur when they adopt AT [54]. To date, 12 reviewed articles have investigated this domain using 4 instruments.

##### Psychosocial Impact of ADs Scale

The Psychosocial Impact of Assistive Devices Scale (PIADS) scale was developed for evaluating AT devices, asking patients how their life has been affected by using the AT equipment [86]. It is the most used scale for evaluating psychosocial impact; indeed, 9 articles used this instrument, showing that this tool has been administered to several clinical conditions and AT products. First, studies showed that PIADS has been used for a broad spectrum of clinical conditions in need of APs: diseases of the nervous system (eg, spastic cerebral palsy) and sense organs (eg, low vision) [47,54,55], severe motor disabilities [32], and traumatic damage to the spinal cord [26]. With regard to APs, PIADS can be applied to 4 main categories of ADs: mobility (eg, wheelchairs and seating systems) [26,54], communication and information management [47,55], domestic activities [56], and controlling devices [32,57].

##### Further Psychosocial Impact Scales

Each of the other 3 instruments was cited by only one article to evaluate specific psychosocial impact components from the patient’s perspective: self-esteem, personal well-being, and self-determination. These instruments have been used with specific categories of patients. For example, the *Rosenberg Self-Esteem Scale* has been used to explore the differences between manual and power wheelchair users regarding self-esteem in persons with SCI [42]. The *Arc’s Self-Determination Scale* allowed for investigating autonomy, self-regulation, psychological empowerment, and self-realization in young patients with cerebral palsy using argumentative and alternative communication APs for at least 15 years [36]. Finally, *Personal Wellbeing Index* ensures well-being assessment in community-dwelling people (aged >65 y) who use APs to achieve individual goals for safety and security at home [58].

#### Caregiver Burden

Care activities can directly and indirectly impact caregivers’ health and life [20]. AT can reduce users’ dependence on human care, especially assistance from informal caregivers (ie, friends, family, and partners). In fact, it has been shown that the use of AT can decrease the caregivers’ physical and psychological burden (eg, stress and anxiety) [37]. However, AT may not completely eliminate responsibility and stress. Although ATs increase caregivers’ sense of freedom and independence, some ADs may require their support. Therefore, it is essential to study caregivers’ burden associated with AT [20].

Overall, 6 articles have focused on evaluating the impact of AT interventions on the burden experienced by informal caregivers. In detail, 2 instruments were used.

#### CATOM Tool

In total, 5 reviewed studies involved caregivers of APs users to investigate their burden through the CATOM questionnaire. CATOM is a tool that can provide a comprehensive evaluation of the burden experienced by informal caregivers, especially psychological burden [59]. Most papers (4/5, 80%) addressed caregivers of patients with motor disabilities who needed APs for activities related to personal mobility, above all powered wheelchairs [20,21]. However, one study showed that CATOM can be used to assess caregiver burden involving a wider range of clinical conditions [37]. Only one research group adopted CATOM in the assessment of caregivers of users of products for domestic activities and participation in domestic life [24].

#### Zarit Burden Interview

Zarit Burden Interview (ZBI) is a caregiver self-report measure used to evaluate the burden in supporting people needing ATs [87]. To date, 1 study has used this tool to evaluate the burden of caregivers of people with dementia using APs for performing domestic activities [44].

#### Quality of Life

##### Overview

The WHO defines quality of life as an individual’s perception of their position in life in the context of the culture and value systems in which they live and in relation to their goals, expectations, standards, and concerns. It has a multidimensional nature and can be influenced by several factors and events that occur in a person’s life. To date, 8 reviewed articles have investigated this domain using 6 instruments.

##### Short Form Health Survey

Overall, 3 studies adopted the 36-item Short Form Health Survey (SF-36) to investigate patient-perceived quality of life in participants using APs [88]. SF-36 allows for evaluating 8 domains of quality of life that could be influenced by the prescribed APs: physical functioning, energy or vitality, bodily pain, general health perceptions, limitations owing to physical and emotional problems, social role functioning, and mental health or emotional well-being. The reviewed article is heterogeneous in terms of APs and the clinical population. The APs belong to the mobility category, including wheelchairs and compressive short-sleeve jackets. As clinical populations, these studies involved patients with hereditary pathologies of the connective tissue, specifically Ehlers-Danlos syndrome [60] or SCI [26].

##### Further Quality of Life Scales

Each of the other 5 instruments was cited by only 1 article to evaluate the quality of life of patients with several clinical conditions and their caregivers. For example, (1) the *EuroQoL-5D-5L* is an instrument that evaluates the participant’s perception of quality of life, investigating 5 dimensions: mobility, self-care, usual activities, pain or discomfort, and anxiety or depression. One study involving 495 patients with dementia used this tool to evaluate the quality of life in patients with ADs for domestic activities and participation in domestic life and their caregivers [44]. A study by Londral et al [40] described and used 2 questionnaires on quality of life to evaluate the impact of early support with assistive communication devices in patients with amyotrophic lateral sclerosis and their caregivers. First, (2) the *McGill Quality of Life Questionnaire* is used as a self-reported multidimensional tool to investigate patients’ and their caregivers’ overall quality of life, including physical well-being, psychological symptoms, existential well-being, and support. The other scale, (3) *World Health Organization Quality of Life (WHOQOL) brief version* [89], is administered only to caregivers to deepen the quality of life in 4 domains: physical and psychological health and well-being, social relations, and environment. This questionnaire has also been used with wheelchair users to investigate how the provision of wheelchairs affects their quality of life domains compared with wait-listed controls [22]. Another tool used with patients who needed assistive communication devices is the (4) *Quality-of-Life Profile for People with Physical and Sensory Disabilities* (QOLP) [90]. QOLP allows for collecting the perspective and experience (eg, thoughts, feelings, beliefs, attitudes, and resources) of people with disability, particularly young men who have used the assistive communication system for at least 15 years [36]. Finally, the (5) *Assessment Quality of Life* [91] was used to evaluate 8 dimensions of quality of life (ie, independent living, pain, senses, coping, mental health, happiness, relationships, and self-worth) in community-dwelling people (aged >65 y) who use APs for domestic activities and participation in domestic life [58].

#### Participation

##### Overview

For many people with disabilities, access to ATs has been identified as a facilitator of the full enjoyment of human rights and participation in society and employment [22]. Overall, 7 articles have focused on evaluating the impact of AT interventions on the participation domain. In detail, 5 instruments were used. However, only 2 instruments have been created to evaluate participation using AT, specifically mobility APs: the Nordic Mobility-Related Participation Outcome Evaluation of Assistive Device Intervention (NOMO) [61] and the Assistive Technology Outcomes Profile for Mobility (ATOP-M) [92].

##### NOMO Tool

The NOMO instrument evaluates the effectiveness of mobility devices in assessing mobility-related participation [61]. To date, only 1 study has used NOMO as an AT outcome measure involving patients with different self-reported impairments who received a powered mobility device (powered wheelchair and scooter) [61].

##### ATOP-M Tool

The ATOP-M is a self-report measure of the impact of mobility devices on the level of activity and participation of the user [92]. Mortenson et al [59,62] conducted 2 studies involving a wide user base (>100 patients for the study) in which they proposed the ATOP-M as a valid instrument to evaluate the impact of power wheelchairs on the level of participation.

##### Late Life Disability Index

The Late Life Disability Index (LLDI) measures participation frequency and perceived limitation in 16 life tasks [93]. It has been frequently used as an outcome measure in geriatric research. The 3 articles that involve this scale converge in the evaluated APs, that is, powered wheelchairs. On the contrary, the studies disagree on the target. Indeed, 2 studies focused on patients. One study involving 115 end users underlined that the original scale might not be applicable to all power wheelchair users [62]. A following study by Mortenson et al [24] supported the use of LLDI in wheelchair users with multiple sclerosis and SCI. Finally, in the last study, the authors showed that LLDI can be successfully administered to caregivers, collecting the patients’ limitations from the caregivers’ point of view [20].

##### Craig Handicap Assessment and Reporting Technique

The Craig Handicap Assessment and Reporting Technique (CHART) scale assesses the degree of social and community participation [94]. It is designed as an interview tool, administered face-to-face or via telephone. Two reviewed studies proposed CHART as a tool for evaluating social participation related to manual and powered wheelchairs [22]. In addition, Hastings et al [42] used this interview with patients with tetraplegia caused by SCI.

### Confidence

The trust of AT users that products work properly is subjective but also depends on device reliability.

To date, only 1 questionnaire has been implemented and used to evaluate this outcome. In detail, 6 reviewed studies have focused on the evaluation of confidence using the *WheelCon-P,* a self-report questionnaire that measures confidence with manual or power wheelchair use [95]. This tool has been administered to wheelchair users affected by several clinical conditions, particularly neurodegenerative conditions, such as autosomal recessive spastic ataxia (2 studies [23,38]), multiple sclerosis, and traumatic damage to the spinal cord [59].

### User Experience: Usability and Acceptability

#### Overview

Usability is defined in the standard “ISO 9241-11:2018 Ergonomics of human-system interaction—Part 11: Usability” as “relevant when designing or evaluating interactions with a system, product or service.” Moreover, usability is “relevant to the use by people with the widest range of capabilities,” and it is defined as “the extent to which a system, product or service can be used by specified users to achieve specified goals with effectiveness, efficiency and satisfaction in a specified context of use.” “Acceptability” can be considered a higher level concept compared with usability and serves as a trade-off among all factors affecting the adoption of new technologies [96].

Furthermore, 3 articles focused on the user experience evaluation involving questionnaires assessing the usability and acceptability of the prescribed ADs. Specifically, 3 instruments were used.

#### System Usability Scale

The System Usability Scale (SUS) is a valid, reliable, and short questionnaire used to evaluate the overall usability of a wide range of technological devices [97]. However, only one study used SUS to evaluate ADs [32]. This study involved patients with severe motor disabilities who required a smart environment controlled by infrared oculography.

#### AT Outcome Measure

One study introduced the Assistive Technology Outcome Measure (ATOM) as a practical clinical tool that assesses AT usability and service in a short, easy-to-administer manner. This study showed the capability of ATOM to be administered to several clinical populations experienced in using a specific device. However, to date, it has only been used to evaluate the usability of APs for activities and participation related to personal mobility and transportation, specifically wheelchairs and seating systems [43].

#### Service User Technology Questionnaire

One study introduced the self-report questionnaire Service User Technology Questionnaire (SUTAQ) to evaluate the acceptability of ADs and identify the characteristics of people who were likely to reject technological health services [44]. The study involving 495 patients showed that SUTAQ can be easily administered to patients with dementia, which could have cognitive limitations [44]. In this context, the AT outcome measure has been used to evaluate the acceptability of ADs for domestic activities and participation in domestic life, which play an important role in increasing the quality of life of patients with neurodegenerative diseases and their caregivers.

#### Further Domains

It is well known that AT interventions can reduce the burden and psychological distress in the families of patients who need APs [44]. Among the behavioral symptoms that could affect the well-being and quality of life of caregivers, anxiety and depression symptoms were investigated. Gathercole and Howard [44] investigated psychological distress in caregivers of patients with dementia who needed APs for performing domestic activities. In detail, the caregivers underwent an evaluation with the S*tate–Trait Anxiety Inventory-6 items* [98] for anxiety symptoms and the screening tool *Centre for Epidemiologic Studies Depression Scale Revised* for depressive symptoms [99]. In another study, Rushton et al [20] evaluated the anxiety and depression symptoms of informal caregivers of powered wheelchair users using a single self-assessment scale, *Hospital Anxiety and Depression Scale*.

### To Which Categories of AT Have the Available Tools Been Applied?

#### Overview

The ISO 9999:2016 standard classification has been used to define the AT categories. The studies involved in this review describe tools applied to 4 AT categories (Multimedia Appendix 3).

The category *APs for activities and participation relating to personal mobility and transportation* (ISO class 12) was the most involved in the reviewed article, being studied in 32 papers (32/50, 64%). The other 3 categories, *APs for communication and information management* (ISO class 22; 8/50, 16%), *APs for domestic activities and participation in domestic life* (ISO class 15; 6/50, 12%), and *APs for controlling, carrying, moving, and handling objects and devices* (ISO class 24; 2/50, 4%) were involved in fewer studies.

#### 12 APs for Activities and Participation Relating to Personal Mobility and Transportation

Products are intended to support or replace a person’s capacity to move indoors and outdoors (ie, walking), transfer from one place to another, or use personal or public transportation.

Overall, 32 articles underlined the importance of APs for mobility and introduced several tools that could be used as AT outcome measures. This heterogeneous category involves manual and powered wheelchairs (the most used AP, cited in 28/32, 88% of papers), scooters, canes, crutches, or walkers. The 29 tools used in the reviewed articles allowed for assessing 7 domains, including functional efficacy (14/29, 48%), participation (5/29, 17%), satisfaction (4/29, 14%), psychosocial impact (2/29, 7%), quality of life (2/29, 7%), usability (1/29, 3%), and confidence (1/29, 3%). The functional efficacy was assessed by all papers involving APs for mobility using 14 tools. Of these instruments, 7 instruments are specifically designed and developed for evaluating APs (ie, WST-Q, WST, SCI-FI or AT, FEW, FMA, WUSPI, and IPPA), of which 5 (71%) are for mobility (WST-Q, WST, FEW, FMA, and WUSPI). The most commonly used questionnaire is the WST-Q, a standardized evaluation method developed and used to self-evaluate manual or powered wheelchair capacity, confidence, and performance in 32 power mobility skills [23,59]. The WST-Q is an effective outcome measure for evaluating functional efficacy in all wheelchair users [22,59], including patients with neurodegenerative and traumatic conditions [20,21,23,25,38,59,62]. In addition, 3 studies reported the WST as a reliable tool for evaluating the mobility capacity of manual wheelchair users [25-27]. Other tools allowed for evaluating specific subdomains of functional efficacy. First, 6 studies focused on changes in patients’ functional independence in performing activities of daily living [29]. In detail, 1 study used FIM and its modified version Self-Report Functional Measure to deepen changes in the patient’s level of disability in carrying out everyday motor tasks [29], whereas 3 studies adopted the FEW and its adapted version FMA to evaluate performance in personal care and daily routine tasks. Unlike the FEW, which is designed only for wheelchairs [34], the FMA includes items relevant to individuals who use any mobility devices, such as wheelchairs, scooters, canes, crutches, or walkers [22,33]. Other valuable tools cited to evaluate the outcome of an AT intervention with manual and powered wheelchairs on a person’s functional performance in performing everyday activities and mobility are the OTFAC [43], Barthel Index [38], and Spinal Cord Independence Measure 3 [42]. Three studies have used Life-Space Assessment to quantify power mobility during the last 4 weeks involving patients who need mobility APs, including mobility service dogs [26] and manual and powered wheelchairs [38,59]. With regard to tools for assessing difficulty in carrying out everyday tasks, the articles proposed the questionnaire COPM [26,34], the Life-H [38], and a scale designed for evaluating AT intervention, IPPA [37]. Finally, 3 studies used SCI-FI or AT to assess mobility ADs outcome in patients with SCI [28,29], focusing on 4 domains: basic mobility, self-care, fine motor function, and ambulation [31]. Moreover, WUSPI was used in a study involving this clinical category as a self-report outcome measure to evaluate the effect of mobility service dogs on shoulder pain in wheelchair users during functional activities [37]. Regarding satisfaction, 4 tools were used to evaluate this aspect in patients needing APs for mobility. Among the 11 articles investigating satisfaction, most (n=5, 45%) have used QUEST 2.0 [26,34,60,63,64], followed by WhOM (n=4, 36%) [38,45,51,52], WSQ (n=1, 9%) [53], and Life-H (n=1, 9%) [37]. Unlike QUEST 2.0 and Life-H, WhOM and WSQ are instruments designed for wheelchairs and seating systems intervention. With regard to the participation domain, changes in the degree of social participation (including perceived limitations) related to manual and powered wheelchairs were assessed in 7 articles by using 4 different tools: CHART [22,42], NOMO 1.0 [61], LLDI, and ATOP-M [59,62]. Among them, 2 instruments, NOMO and ATOP-M, have been created to evaluate participation changes related to assistive mobility [61,92]. Moreover, LLDI was successfully administered to caregivers, collecting the patients’ limitations from the caregivers’ point of view [20]. Furthermore, 1 study investigated the usability of wheelchairs and seating systems using the short tool ATOM [43]. WheelCon-P is the only questionnaire implemented and used for evaluating the confidence domain [62] and is designed for being administered to manual and powered wheelchair users. To date, 6 studies converged in supporting that it is usable with many neurodegenerative and traumatic clinical conditions [23,25,38,59,62,65]. The psychosocial impact was assessed by 5 articles using PIADS [26,34,43,54] and Rosenberg Self-Esteem Scale [42], whereas changes in quality of life because of APs (ie, wheelchairs, mobility service dogs, and compressive short-sleeve jackets) were evaluated in 4 articles through SF-36 [22,26,60,64] and in a single article by WHOQOL brief version [22]. Finally, 4 reviewed studies have involved caregivers of APs for mobility users to investigate their perceived psychological burden through the CATOM questionnaire [20,37,41]. Moreover, Rushton et al [20] deepened the anxiety and depression symptoms of informal caregivers of powered wheelchair users using the self-assessment Hospital Anxiety and Depression Scale.

#### 15 APs for Domestic Activities and Participation in Domestic Life

Products are intended to support or replace a person’s capacity to carry out domestic and everyday actions and tasks, including preparing or eating food and household cleaning.

Overall, 7 reviewed articles were concerned with APs for domestic activities, such as plastic cap wrenches, key turners, and AP for writing. To date, the 12 used tools allowed for assessing 5 domains, functional efficacy (3/12, 25%), satisfaction (2/12, 17%), psychosocial impact (2/12, 17%), quality of life (2/12, 17%), caregiver burden (2/12, 17%), and usability (1/12, 8%). In detail, 3 articles assessed satisfaction using QUEST 2.0 [46,49] and Life-H [37]. Only 1 article evaluated the usability of APs regarding the acceptability of ADs [44]. The psychosocial impact was assessed using PIADS [56] and Personal Wellbeing Index [58], whereas the quality of life was evaluated in 2 articles using Assessment Quality of Life [58] and EuroQol-5 [44]. Moreover, 3 articles investigated the functional efficacy domain, focusing on changes in primary and instrumental activities of daily living using BADLS [44], IPPA [37], and COPM [58]. Finally, CATOM and ZBI were used in assessing the burden of caregivers of people with a wide range of clinical conditions (including dementia) who use APs for performing domestic activities [37,44]. Moreover, Gathercole and Howard [44] investigated the psychological distress in caregivers of patients with dementia using State–Trait Anxiety Inventory for anxiety symptoms and Centre for Epidemiologic Studies Depression Scale Revised for depressive symptoms.

#### 22 APs for Communication and Information Management

Products are intended to support or replace a person’s capacity to receive, send, produce, and process information differently, including communicating through language, signs, and symbols; receiving; generating messages; carrying on conversations; and using communication devices.

Overall, 8 articles focused on this category, including several APs, such as pillbox, head-mounted visual AT, augmentative and alternative communication systems, speech synthesizers, and electric calendars. The 15 tools used in the reviewed articles allowed for assessing 5 domains, functional efficacy (n=9, 60%), quality of life (n=3, 20%), psychosocial impact (n=2, 13%), and satisfaction (n=1, 7%). In detail, the 4 articles evaluating satisfaction have used QUEST 2.0 [35,47,48,66]. The psychosocial impact was assessed by 3 articles using PIADS [47,55] and Arc’s Self-Determination Scale [36], whereas the quality of life was evaluated in 2 articles through McGill Quality of Life Questionnaire, WHOQOL brief version [40], and Quality of Life Profile [36]. Finally, the functional efficacy of APs has been investigated by 4 articles in terms of (1) functional changes in primary and instrumental activities of daily life with Amyotrophic Lateral Sclerosis Functional Rating Scale [40] and Life-H [35]; (2) global cognitive abilities with Neurobehavioral Cognitive Status Examination, Dementia Behavior Disturbance Scale, and Mini-Mental Examination State [39]; (3) language domain—FACS-A [35,36] and Communicative Effectiveness Index modified [40] for functional communication; Gray Silent Reading Test for reading comprehension ability; Test of Auditory Comprehension of Language, revised for comprehension of semantics, morphology, and syntax; and Peabody Picture Vocabulary Test, revised for receptive (hearing) vocabulary [36]. To date, no studies have evaluated the usability and confidence involving patients needing APs for communication and information management or the burden of their caregiver.

#### 24 APs for Controlling, Carrying, Moving, and Handling Objects and Devices

Products are intended to facilitate a person’s task performance require the movement or manipulation of an object. Two articles focused on this category, including smart environment [32] and environmental control APs (ie, electronic aids for daily living) [57]. The 5 tools used allowed for assessing 4 domains, functional efficacy (2/5, 40%), satisfaction (1/5, 20%), usability (1/5, 20%), and psychosocial impact (1/5, 20%). Sime and Bissoli [32] evaluated the satisfaction and usability of patients with motor difficulties in using smart environments by QUEST 2.0 and SUS, respectively [32]. Moreover, the authors assessed the functional efficacy using FIM and COPM to identify changes in patients’ level of disability and everyday issues owing to AT intervention. Finally, both reviewed articles used PIADS to evaluate psychosocial impact.

#### Further Interesting Insights

Finally, this paragraph focuses on 4 other elements—original purpose of the tools, cross-sectional outcome measures, study participants and administration time, and modality of the instruments.

##### Original Purpose of the Tools

Among the reviewed tools, 17 scales were designed to evaluate the ADs, including the QUEST 2.0, ATD-PA, WhOM, WSQ, ATOM, WheelCon-P, PIADS, IPPA, WST, SCI-FI or AT, FEW, FMA, WST-Q, WUSPI, NOMO 1.0, ATOP-M, and CATOM. These tools allowed the assessment of 7 domains, satisfaction (4/17, 24%), usability (1/17, 6%), confidence (1/17, 6%), psychosocial impact (1/17, 6%), functional efficacy (7/17, 41%), participation (2/17, 12%), and caregiver burden (1/17, 6%).

##### Cross-Sectional Outcome Measures

Approximately half (23/50, 46%) of the reviewed studies described outcome measures applied to several APs in a single category. In contrast, only 2 studies used the same instruments for assessing AT products of multiple AT categories (ie, mobility and domestic activities). Interestingly, these studies involved only the caregivers of AT users (eg, CATOM or IPPA).

##### Participants

Overall, 74% (39/53) of the studies involved small samples of ≤100 patients or caregivers, of which 18% (7/39) involved ≤10 patients (2 case studies). Most studies (44/50, 88%) involved patients with different clinical conditions; 4 articles recruited caregivers, and the other 2 included both categories. Most studies did not report the specific pathological conditions of the sample, including “AT users.” Among the reported clinical conditions, most studies involved patients with SCI (n=8), followed by neuromuscular or musculoskeletal disorders and diseases of the nervous system and sense organs (eg, amyotrophic lateral sclerosis, dementia, stroke, multiple sclerosis, low vision, and autosomal recessive spastic ataxia).

##### Administration Time and Modality of the Instruments

Most instruments were self-report questionnaires completed by the participants involved in the study. Only 8 studies reported objective outcome measure instruments administered by clinicians. Of these, 4 (50%) also reported self-reported measures. With regard to administration time, only 6 studies reported this information.

## Discussion

### Overview

This review was designed to provide an overview of AT outcome measures to try to answer a wide research question: “What AT outcome measures are currently available and used for evaluating individual AT interventions?” It is well known that >1 billion people globally need ≥1 AT, a number that is expected to double by 2050 [1]. Measuring outcomes is necessary to understand the benefits of AT and create evidence-based policies and systems to improve universal access to it [1]. This review aims to provide a critical synthesis of the available and used instruments for AT outcome assessment.

The 50 reviewed articles have shown a wide increase (almost tripled compared with the previous decade) of the studies on outcome measure assessment in the last 10 years. This sudden increase in studies in this area could depend on the numerous initiatives carried out by the WHO and the Global Alliance of Assistive Technology Organizations [1,8].

To date, 53 instruments have been used to assess the outcomes of AT interventions. Despite the many reviewed tools, only 17 scales (approximately 30%) were designed to evaluate specifically the ADs, such as QUEST 2.0, ATD-PA, WhOM, WSQ, ATOM, WheelCon-P, PIADS, IPPA, WST, SCI-FI or AT, FEW, FMA, WST-Q, WUSPI, NOMO 1.0, ATOP-M, and CATOM. Among them, most are specifically designed and developed for manual and powered wheelchairs. It could depend on the fact that mobility APs are the second most used AT category, after glasses, 150 million people in the world need mobility APs, of which 75 million need wheelchairs [1]. The remaining instruments were primarily intended to measure the outcomes of individual intervention programs, which may or may not include APs. Interestingly, 34 instruments were only mentioned once to denote poor uniformity and concordance in the instruments to be used, limiting the possibility of comparing the results of the studies.

### Points of Reflection on Available Tools According to AT Outcome Domains

The identified measures addressed 8 AT outcome domains: functional efficacy, satisfaction, psychosocial impact, caregiver burden, quality of life, participation, confidence, and usability. Functional efficacy is the most evaluated intervention outcome being consistent with the primary goal of AT interventions to achieve an enhancement in everyday functioning. Moreover, 26 scales were used to obtain outcome data within this domain, of which 6 (23%) instruments were specifically designed and developed for evaluating APs. The most commonly used questionnaire is the WST-Q [67]. The type of constructs potentially evaluated with these tools is widely heterogeneous. First, they allow for measuring changes in functional independence in basic (eg, eating and dressing) and instrumental (eg, house cleaning and hobbies) daily living activities [29,37,44]. Moreover, a wide number of tools focus on improvement in ambulation and wheelchair mobility, including changing or maintaining body positions and transfers [23,26]. Finally, changes in the language domain—receptive language skills, reading comprehension, and functional communication (eg, understanding television and radio)—were investigated [36,40].

Despite the importance of functional efficacy, it is well known that the positive impact of APs goes far beyond functional efficacy by positively affecting the well-being, quality of life, participation, and the inclusion of individual users—families [1,2]. In this context, several articles have shown that numerous AT outcome assessment measures allow for evaluating the impact of AT intervention’s on psychosocial impact, quality of life, and caregiver burden. The psychosocial impact was investigated using 4 instruments that analyzed subjective perceptions of changes in competence, adaptability, self-esteem, and self-determination. The most commonly used questionnaire is PIADS [47], which is used with a broad spectrum of neurological and traumatic clinical conditions [47,54,55]. The quality of life of APs users has been investigated using 6 different tools that provide information on the status and any changes in physical functioning, energy or vitality, bodily pain, general health, social role functioning, and mental health or emotional well-being. Moreover, the reviewed articles showed that other tools, such as CATOM and ZBI, can be used to evaluate the quality of life of caregivers, focusing on their psychological burden [40]. Evaluating this aspect appears to be a priority as care activities can, directly and indirectly, affect caregivers’ health and life [20], and assistive solutions can hopefully lower the caregiver burden.

In addition to quality of life, some studies have focused on participation in society [22]. To this end, 5 instruments were used to investigate the frequency and perceived limitation of participation in several everyday tasks [61], such as visiting friends and family in their homes, participating in active recreation, going to restaurants, and shopping for groceries. The International Classification of Functioning supported the importance of participation as a critical part of psychosocial well-being [100,101]. However, only a few articles (8/50, 16%) have focused on this domain. Further studies should be conducted to elucidate this component.

Despite the need for AT and its potential role in improving patients functioning and quality of life, over the years, several studies have focused on the high rate of abandonment of the received AP owing to the poor performance of the device, which does not meet the environmental needs and does not consider the opinion of the user [6,22]. In this context, evaluating satisfaction with using APs appears to be a priority because giving users a quantifiable voice in APs functioning could lead to better design and provision and increased use [53]. The review showed that 5 tools were available and used, and 56% (28/50) of the articles focused on the satisfaction domain. Similarly, evaluating the user experience of patients in using APs in terms of technological usability and acceptability appears important. However, only 3 articles and 3 tools (SUS, ATOM, and SUTAQ) have focused on this domain. It is important to keep in mind that data on needs, barriers to access, and users’ experience with APs are equally crucial to guide the design of appropriate systems to meet reported needs [1].

### Points of Reflection on Available Tools According to AT Category

The review showed that approximately half of the reviewed studies described outcome measures applied to several APs, but in a single category. In contrast, only 2 studies used the same instruments for assessing AT products of multiple AT categories (ie, mobility and domestic activities). Interestingly, these studies involved only the caregivers of AT users. Moreover, the number of studies and tools used for each category of APs was not homogeneous. In detail, the most evaluated category is APs for activities and participation relating to personal mobility and transportation. The 29 used tools allow for assessing all 8 AT outcome domains. It is important because among 1 billion people needing APs, approximately 150 million need mobility aids, of which 75 million need wheelchairs and 35 prostheses or orthoses. In contrast, few tools are used as outcome measures for an intervention involving APs for controlling, carrying, moving, and handling objects and devices [32]. With regard to APs for domestic activities and communication, few studies have been conducted, but the available and used tools have evaluated several domains, such as functional efficacy, satisfaction, psychosocial impact, quality of life, and caregiver burden. Further studies are needed to investigate these domains.

### Further Interesting Insights

Another interesting point of reflection is that 74% (39/53) of the studies involved small samples of ≤100 patients or caregivers, of which 18% (7/39) involved ≤10 patients (with 2 case studies). Most studies (44/50, 88%) involved patients with different clinical conditions; 4 articles recruited caregivers and the other 2 included both categories. Most studies did not report the specific pathological conditions of the sample involving “AT users.” Indeed, a global report on AT supported that the sample of those who need AT is widely heterogeneous, including people with communicable and noncommunicable, mental health, neurodegenerative (eg, amyotrophic lateral sclerosis, dementia, stroke, multiple sclerosis, low vision, and autosomal recessive spastic ataxia), or traumatic conditions (eg, SCI) [1].

Finally, an open point is the administration time and modality of the instruments as most instruments are self-report questionnaires filled in by the participants, and the administration time is almost never reported.

### Implications for Clinical Practice

To our knowledge, this is the first systematic review to investigate the available outcome measures for evaluating individual AT interventions. Owing to the growing need for standardized methods to track individual AT interventions [9], this work could represent a good guide for promoting the use of validated AT outcome measures in clinical practice that can be helpful to AT assessment teams in their everyday activities and the improvement of clinical practice.

## Appendix

Multimedia Appendix 1 Graphical representation of the trend in the number of publications on outcome measure assessment over time.

Multimedia Appendix 2 Information (evaluated domain, assistive technology [AT] category, description, and scoring) on available instruments was used to provide an AT outcome measure assessment.

Multimedia Appendix 3 Evaluable domains for each category of assistive technology with the available tools.

Multimedia Appendix 4 PRISMA Checklist.

## Abbreviations

AD: assistive device
AP: assistive product
AT: assistive technology
ATD-PA: Assistive Technology Device Predisposition Assessment
ATOM: Assistive Technology Outcome Measure
ATOP-M: Assistive Technology Outcomes Profile for Mobility
BADLS: Bristol Activity of Daily Living Scale
CATOM: Caregiver Assistive Technology Outcome Measure
CHART: Craig Handicap Assessment and Reporting Technique
COPM: Canadian Occupational Performance Measure
FACS-A: Functional Assessment of Communication Skills for Adults
FEW: Functional Everyday with a Wheelchair
FI: Functional Index
FIM: Functional Independence Measure
FMA: Functional Mobility Assessment
IPPA: Individually Prioritised Problem Assessment
LIFE-H: Assessment of Life Habits
LLDI: Late Life Disability Index
NOMO: Nordic Mobility-Related Participation Outcome Evaluation of Assistive Device Intervention
OTFAC: Occupational Therapy Functional Assessment Compilation
PIADS: Psychosocial Impact of Assistive Devices Scale
PRISMA: Preferred Reporting Items for Systematic Reviews and Meta-Analyses
QOLP: Quality-of-Life Profile for People with Physical and Sensory Disabilities
QUEST 2.0: Quebec User Evaluation of Satisfaction with Assistive Technology
SCI: Spinal Cord Injury
SF-36: 36-item Short Form Health Survey
SUS: System Usability Scale
SUTAQ: Service User Technology Questionnaire
WheelCon-P: Wheelchair Use Confidence Scale for power wheelchair users
WHO: World Health Organization
WhOM: Wheelchair Outcome Measure
WHOQOL: World Health Organization Quality of Life
WSQ: Wheelchair Satisfaction Questionnaire
WST: Wheelchair Skills Test
WST-Q: Wheelchair Skills Test Questionnaire
WUSPI: Wheelchair User’s Shoulder Pain Index
ZBI: Zarit Burden Interview
