# Supplementary material for: Available Assistive Technology Outcome Measures: Systematic Review
Source: JMIR Rehabil Assist Technol. 2023 Nov 15;10:e51124. doi: 10.2196/51124 (PMC10687703; doi:10.2196/51124)
Supplement: Multimedia Appendix 1 [file rehab_v10i1e51124_app1.docx]

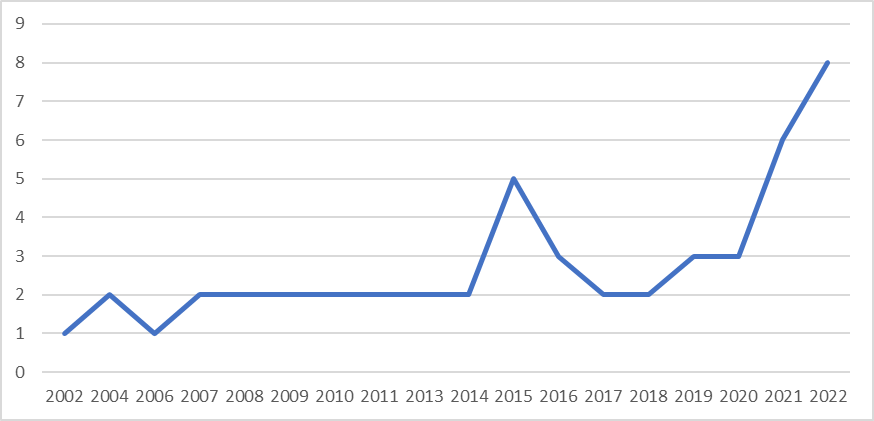


Multimedia Appendix 1. Graphical representation of the trend in the number of publications on outcome measures assessment over time
