# Supplementary material for: Available Assistive Technology Outcome Measures: Systematic Review
Source: JMIR Rehabil Assist Technol. 2023 Nov 15;10:e51124. doi: 10.2196/51124 (PMC10687703; doi:10.2196/51124)
Supplement: Multimedia Appendix 2 [file rehab_v10i1e51124_app2.docx]

| **Outcome Domain** | **Measurement**  **Tool** | **AT Category** | **Outcome** | **Scoring** | **Population (patients/caregiver)** |
| --- | --- | --- | --- | --- | --- |
| *Satisfaction* | QUEST 2.0 | 12-15-22-24 | Individual’s satisfaction with the assistive device and the related services | 12 questions on 5-point Likert scale from 1 (not satisfied at all) to 5 (very satisfied). Three scores: Device subscale (eight questions), Service subscale (four questions), a composite score | severe dysarthria (10; Laffont et al., 2007), hypermobile subtype of Elhers-Danlos syndrome (36; Chaléat-Valayer et al., 2022), intellectual and developmental disabilities (10; Boccardi et al., 2022), low vision (57; Lorenzini et al., 2021), rheumatoid arthritis (1; Janson et al., 2020), motor disabilities (6; Sime et al., 2021), deafness (15; Vincent et al., 2008), pillbox users (14; Schwartz et al., 2020), mobility device users (10; Giesbrecht et al., 2009), 60+ users of seating and mobility systems (30; Trefler et al., 2004), older adults users of MWC or PWC (132; Karmarkar et al., 2009); MWC users with SCI (24; Vincent et al., 2019), |
|  | LIFE-H | 12-15-22 | Individual’s satisfaction regarding the accomplishment of life habits | 18 items on 5-point Likert scale from 1 (very unsatisﬁed) to 5 (very satisfied) | AT users' caregivers (88; Mortenson et al., 2013) |
|  | ATD-PA | - | Consumer’s subjective satisfaction in several functional areas when using a particular AT | device form- self-assessment questionnaire: 12 items on 5-point Likert scale | AT users (115; Koumpouros et al., 2017) |
|  | WhOM | 12 | Satisfaction with performance of self-identified activities while using their wheelchair | 11-point scale: 0 (Not at all important/satisfied) to 10 (Extremely important/satisfied). Total scores: mean satisfaction (MeanSat;/number of activities), and mean importance x satisfaction | MWC and PWC users with ARSA (36; Bourassa et al., 2020), community living volunteers with SCI (50; Miller et al., 2011), SCI (51; Rushton et al., 2010), residents in long-term care (55; Parvaneh et al., 2014) |
|  | WSQ | 12 | Explicit aspects of user satisfaction with wheelchair function | 16 visual analogue scale questions: A (Excellent) to E (Poor) + qualitative comment. | MWC users (30; Bane et al., 2021) |
| *User Experience* | SUS | 24 | Usability - "easy-to-use" and "easy-to-learn" | 10 items on 5-point Likert scale (from "Strongly disagree" to "Strongly agree". Total score (range: 0-100) = sum of the item scores * 2.5 | motor disabilities (6; Sime et al., 2021) |
|  | ATOM | 12 | Usability - 7 domains: use and community; comfort; hassle; self-perceived assessment of function; assistance and burden of care; service satisfaction; user’s knowledge of AT resource. | 19 items on 4-point Likert scale to measures 7 construct | wheelchair and seating system users (31; Harris et al., 2008) |
|  | SUTAQ | 15 | Acceptability of Assistive device - domains: enhanced care; increased accessibility; privacy and discomfort; care personnel concern; kit as substitution; satisfaction | 22 items on 6-point Likert scale, reflecting agreement with the item statements | dementia (495; Gathercole et al., 2021) |
| *Confidence* | WheelCon-P | 12 | power mobility users’ self-efficacy (confidence) in five areas: negotiating the physical environment, wheelchair-related activities, problem solving, advocacy and managing social situations | 59 items on a scale ranging from 0 (Low confidence) to 100 (High confidence). The overall confidence score is calculated as the mean score of all items | MWC and PWC users with ARSA (36; Bourassa et al., 2020 + 32; Bourassa et al., 2022), Multiple sclerosis and spinal cord injury/disease (128; Mortenson et al., 2015), experienced MWC users (31 Berardi et al., 2018), PWC users (72; Rushton et al., 2016), power mobility users (115; Mortenson et al., 2014) |
| *Psychosocial Impact* | PIADS | 12-15-22-24 | How patient's life has been affected by AT - 3 domains: competence (feelings about AT impact on their sense of competence, productivity, usefulness, performance, and independence), adaptability (willingness to try new things and queries self-perceived ability to participate) and self-esteem (AT impact on emotional wellbeing) | 26 items on a self-rating 7-point scale ranging from −3 (maximum negative impact) and +3 (maximum positive impact) Three subscores: competency (12 items), adaptability (6 items) and self-esteem (8 items). Each subscore is calculated by the average of responses for the specific items under each concept. | low vision (57; Lorenzini et al., 2021), motor disabilities (6; Sime et al., 2021), neurological conditions (80; Jiménez-Arberas et al., 2021), CP (1; Hsieh et al., 2022), users of EADL (7; Ripat et al., 2004), mobility device users (10; Giesbrecht et al., 2009), MWC users with SCI (24; Vincent et al., 2019),, wheelchair and seating system users (31; Harris et al., 2008), individuals in need of AT (60; Orellano-Colón et al., 2016) |
|  | Arc’s Self-Determination Scale | 22 | Self-determination (autonomy, self-regulation, psychological empowerment, and self-realization) | 72 questions: open-ended questions, questions with Likert scale (0 = I do not even if I have chance; 3 = I do every time I have a chance) and binary choices (agree/don’t agree) | CP CCA AT users (7; Lund et al., 2006) |
|  | PWI | 15 | Personal Well-being - satisfaction in standard of living, health, life achievements, personal relationships, feelings of safety, community integration, future security and overall life. | 8 items on the Likert scale ranging from 1 to 10. Higher scores indicate greater personal well- being | community-dwelling people aged 65+ (30; Gordon et al., 2022) |
|  | RSES | 12 | Self-esteem | 10 statements (5 positive, 5 negative) with 4 levels of agreement (from "Strongly disagree" to "Strongly agree"). Higher scores indicate greater self-esteem | wheelchair users with tetraplegia caused by SCI (30; Hastings et al., 2011) |
| *Quality of life* | EQ-5D-5L | 15 | Patients' perception of quality of life - 5 dimensions (mobility, self-care, usual activities, pain/discomfort, anxiety/depression) and a overall score quality of life | 5 items on 5 levels of disability from “no problems” (level 1) to “severe problems” (level 5). Moreover, a VAS scale records the patient’s self-rated health on a vertical visual analogue scale, ranging from 0 The worst health you can imagine) to 100 (The best health you can imagine). | dementia (495; Gathercole et al., 2021) |
|  | MQoL | 22 | 5 dimensions of quality of life: 2 health- related domains [Physical well-being, physical symptoms] and 3 non-health-related domains [psychological symptoms, existential well-being and support], and overall quality of life | 16 items on a liker scale ranging from 0 to 10. The five domains are calculated by the mean of item scores. A total score was calculated as the mean of all domains: higher scores indicate higher QoL. Moreover, it includes a single-index score which measures the overall quality of life and a section where respondents list what had the greatest impact on their QoL in the past 2 days. | ALS patients and caregivers (44; Londral et al., 2015) |
|  | QOLP-PD | 22 | Quality of life of people with disabilities - nine categories: body and health; thoughts and feelings; my beliefs, attitudes, and values; where I live and spend my time; people around me; (my access to resources, the daily things I do; the things I do for enjoyment, and the things I do to improve myself | 102 items on a 5-point scale for Satisfaction and Importance: 1 (Not at all satisfied/important) and 5 (Extremely satisfied/ important). Scores are derived from a combination of importance and satisfaction. The score ranges from −10 to +10. Scores for each section were averaged to calculate the overall quality of life score. | CP AAC AT users (7; Lund et al., 2006) |
|  | SF-36 | 12 | Patient-perceived health-related quality-of-life - 8 health domains: physical functioning, physical role functioning, bodily pain, social functioning, vitality, mental health, role-emotional and general health. | 36 items dichotomic and Likert scale with 3, 5, 6 points that allow for calculating 8 domains. Scores for each domain are coded, summed, and transformed on to a scale from 0 to 100, with a higher score defining a more favorable health state. Moreover, there is a further single item on changes in respondents' health over the past year. | Elhers-Danlos syndrome (36; Chaléat-Valayer et al., 2022), MWC users with SCI (24; Vincent et al., 2019), 60+ users of seating and mobility systems (30; Trefler et al., 2004) |
|  | WHOQOL-BREF | 12-22 | Quality of life within the context of an individual’s culture, value systems, personal goals, standards, and concerns - 4 domains: physical health, psychological health and well-being, social relations, and environment. | 26 items on Likert scale from 1 (very poor) to 5 (very good). Scores are calculated on a 100-point scale: higher scores correspond to better QoL. | ALS patients and caregivers (44; Londral et al., 2015), wheelchair recipients (344; Toro et al., 2016), mobility ADs users (41; Kumar et al., 2013) |
|  | AQoL-8D | 15 | Quality of life - eight dimensions Independent Living, Pain, Senses, Coping, Mental Health, Happiness, Relationships and Self- worth. | 35 items with four to six levels each. Lower scores indicate greater quality of life | community-dwelling people aged 65+ (30; Gordon et al., 2022) |
| *Functional Efficacy* | WST | 12 | Wheelchair skills - capacity (can do) - three skill level: indoor (e.g., moves controller away and back, turns 90° while moving forward), community (e.g., selects drive modes and speed, and rolls 100m) and advanced (e.g., ascends 5° incline and descends 15cm curb) | Objective measure. Items: Manual (33 items) and Power (32 items) wheelchair test for patients on dichotomous score pass (1)/fail (0). Total capacity score is calculated as = sum of individual skill scores/([number of possible skills – number of NP scores – number of TE scores]) X 100% [NP -wheelchair does not have the parts to allow this skill; TE= testing error]. Safe is evaluated for each skill as safe/unsafe score (using the same formule of capacity). The WST for caregivers is composed of 31 items for MWC and 29 for PWC | wheelchair users (24; Kirby et al., 2002), MWC users with SCI (24; Vincent et al., 2019), PWC users (72; Rushton et al. 2016) |
|  | WST-Q | 12 | Manual or powered wheelchair - capacity (can do) and performance (does do)- - three skill level: indoor (e.g., moves controller away and back, turns 90° while moving forward), community (e.g., selects drive modes and speed, and rolls 100m) and advanced (e.g., ascends 5° incline and descends 15cm curb) | Self-reported measure, administered via semi-structured interview. Items: Manual (33 items) and Power (32 items) wheelchair test or patients on dichotomous score yes(1)/no(0). For each skill, Two scores Capacity and Performance are calculated. for example, C. Can you get yourself and your wheelchair down the stairs?; P. Have you done this in the past month? The Total Scores are calculated as = sum of yes/([number of possible skills – number of NP scores – number of TE scores]) X 100%. Safe is evaluated for each skill as safe/unsafe score (using the same formule of capacity). The WST for caregivers is composed of 31 items for manual and 29 items for power wheelchairs. | informal caregivers of PWC users (35; Rushton et al., 2017), PWC users (72; Rushton et al., 2016), MWC and PWC users with ARSA (36; Bourassa et al., 2020 + 32; Bourassa et al., 2022, Multiple sclerosis and spinal cord injury/disease (128; Mortenson et el., 2015), power mobility users (115; Mortenson et el., 2014), caregivers of patients with motor disability (35; Labbè et al., 2019), wheelchair recipients (344; Toro et al., 2016), mobility ADs users (41; Kumar et al., 2013) |
|  | IPPA | 12-15 | Perceived effectiveness of an AT intervention - self-rated task difficulty | Subjects identify a list of tasks in which there are difficulties (up to seven problematic activities he/she expects to solve thanks to the AT solution). For each task, they rate the level of importance of the task on 5-point scale ( 5 = "Most important") and the level of difficulty on 5-point scale (5 = "Too much difficulty to perform the activity at all). For each task, the subscore is calculated as importance*difficulty and the total score as the sum of all subscores | caregivers of patients with AT (88; Mortenson et al., 2013) |
|  | LIFE-H | 12-22 | Level of difficulty in carrying out 12 life habits and the type of assistance required to carry out the habits (technical assistance, physical arrangements, human assistance). 12 life habits: nutrition, personal care, fitness, communication, housing, mobility, responsibility, relationship, community life, education, employment, recreation | 69 items on 10-point Likert scale ranging from 0 (Too difficult to perform) to 9 (No difficulty and no help). | caregivers of AT users (88; Mortenson et al., 2013), MWC and PWC users with ARSA (36; Bourassa et al., 2020), deafness (15; Vincent et al., 2008) |
|  | COPM | 12-15 | changes in participant's goal (defined at baseline) performance and satisfaction. | outcome measure implemented via a semi-structured interview. 10-point Likert scale to rate each problem based on the level of importance. The five most important problems chosen are rated based on self-perceived performance and satisfaction with performance. Higher follow-up scores indicate improved performance and satisfaction. | community-dwelling people aged 65+ (30; Gordon et al., 2022), motor disabilities (6; Sime et al., 2021), mobility device users (10; Giesbrecht et al., 2009) |
|  | SCIM III | 12 | Performance in activities of daily living and mobility in individuals with spinal cord injury | 17 items and theee subscales: self-care (4 items), breathing and sphincter control (4 items) and mobility (9 items - two parts: one for the bedroom and bathroom and another for internal and external environments). The final score ranges from 0 (worst) to 100 (best) points,with self-care scores ranging from 0-20 and the other two subscale scores from 0-40. | wheelchair users with tetraplegia caused by SCI (30; Hastings et al., 2011) |
|  | BADLS | 15 | Ability to carry out 20 basic and instrumental activities of daily living (e.g., dressing, hygiene, toilet, preparing food/eating, transport, mobility, spatial-temporal orientation, communication, hobbies, finances, shopping, housework, telephone) | 20 items (evaluated 20 activities) on a 4-point scale (from totally dependent to totally independent + not applicable) | dementia (495; Gathercole et al., 2021) |
|  | ALSFRS-R | 22 | Activities of daily living and global function for patients with Amyotrophic Lateral Sclerosis. Total score and two subscores: bulbar function (speech, salivation and swallowing) and upper limb function (handwriting, handling utensils in daily activities dressing) | 12 questions on a 5-point Likert scale: 0 (unable) to 4 (normal ability). The total score was calculated as the sum of scores, ranging from 48 (normal) to 0. The two subscores range from 12 to 0 and were calculated by summing the first 3 questions for bulbar function and 4-6 for upper limb function | ALS patients and caregivers (44; Londral et al., 2015) |
|  | FIM | 12-24 | Level of assistance demanded to perform 13 motor (e.g., eating, grooming, bathing, dressing, toileting, Bladder and bowel management, transfers, walk/wheelchair, stairs) and five cognitive (e.g. language, problem solving and memory) tasks | 18 items on 7-ordinal scale from 1 (total assistance or not testable) to 7 (complete independence), resulting in a total score of 18–126 (higher score corresponds to higher independence). The two subscale scores are calculated as the sum of motor (range 13-91) or cognition (range 5-35) items | traumatic SCI (269; Tyner et al., 2022) |
|  | SRFM | 12 | Need for assistance for basic and instrumental activities of daily living (e.g., moving around indoors and personal hygiene) (modified version of FIM) | 13 items basic ADL, 5 items instrumental ADL, 8 items of disease severity and 1 item of resource utilization; 4-level response categories: 1(total help or never do) to 4 (no extra time or help). Total score (13-52) is the sum of basic ADL items only, with lower scores indicate greater need for assistance | traumatic SCI (269; Tyner et al., 2022) |
|  | LSA | 12 | How far and how often patients have mobilized with or without assistance during the last 4 weeks. 5 Life-Space Levels: Level 1 (indoors), Level 2 (distance beyond the front door as far as the garden boundary or communal hallway), Level 3 (local neighborhood), Level 4 (town or city), Level 5 (beyond the town or city) | The frequency is measured on a 4-point scale, ranging from less than once per week to daily. The independence score (assistance required from a person and/or assistive technology) is measured on a three-point scale (1-1.5-2). A level score is calculated for each level as level*independence*frequency. Life-space mobility score is calculated as the sum of level scores and ranges from 0 (fully restricted mobility) to 120 (Unrestricted mobility). | MWC and PWC users with ARSA (36; Bourassa et al., 2020), Multiple sclerosis and spinal cord injury/disease (128; Mortenson et al., 2015) |
|  | Barthel ADL Index | 12 | Functional independence in 10 activities of daily living (feeding, personal toileting, bathing, dressing, going to toilet, controlling bladder and bowel, moving from wheelchair to bed and returning, moving on a level surface and ascending/descending stairs. | 10 items (10 investigated activities) on a scale ranging from 0 (Dependent) to 5 or 10, or 15 (independent, according to item). The total score ranges from 0 to 100, representing total independence | MWC and PWC users with ARSA (36; Bourassa et al., 2020) |
|  | SMAF | 12 | Functional ability in five different areas: activities of daily living (7 items); mobility (6 items); communication (3 items); mental functions (5 items) and instrumental activities of daily living (8 items). | 29 items on 4 point scale: 0 (independent)‚ -1 (needs supervision)‚ -2 (needs help)‚ -3 (dependent). For each item, resources available to compensate for the disability are evaluated (e.g., neighbour, aides, voluntary) and a handicap score can be deduced ranging between 0 (independent or presence of resource) to -3 (dependent and without resource). The stability of the resources is also assessed. | AT mobility interventions recipient and caregivers (110; Mortenson et al., 2018) |
|  | OTFACT | 12 | Functional performance (personal care activities, home management activities) and environmental assessment | The OTFACT was administered via telephone as a semistructured interview. Responses were categorized as no deficit, partial deficit or total deficit | wheelchair recipients (31; Harris et al., 2008) |
|  | SCI-FI/AT | 12 | Identify how much difficulty patients with SCI have performing tasks with the AT they typically use. | SCI-FI/AT item banks - 47 Basic Mobility items, 35 Fine Motor items, 71 Self-Care items, 29 Ambulation items, and 56 Wheelchair items. Higher scores indicate better functioning. | traumatic SCI (269; Tyner et al., 2022), mobility ADs users with traumatic SCI (460 Jette et al., 2015 + 460; Slavin et al., 2016), persons with traumatic SCI discharged from inpatient rehabilitation (1237; Ni et al., 2022) |
|  | FACS - A | 22 | Functional Communication - 4 domains: social communication, communication of basic needs, reading/writing/number concepts, daily planning. It covers different activities of daily living such as understanding television and radio, responding in an emergency, and using a calendar. Two subscales Communicative Independence and Qualitative Dimensions of Communication (i.e., adequacy, appropriateness, promptness, communicative sharing). | 43 items on a 7-point based on how much assistance was needed to complete the activity from 1 (does not do) to 7 (does independently). Each domain is rated on the basis of a 5-point Scale of Qualitative Dimensions of Communication ranges from 1 (partner carries all communication burden) to 5 (individual and partner share equally in communication). The average scores of each domain/dimension and the overall scores (calculated as average of scores in each domain/dimension) of Communication Independence and Qualitative Dimensions are obtained | CP AAC AT users (7; Lund et al., 2006), deafness (15; Vincent et al., 2008) |
|  | FEW | 12 | Perceived functional independence of wheelchair or scooter users - degree of problems in performing 9 functional tasks (e.g., stability, comfort needs, health needs, operate, reach, transfer, personal care, indoor mobility, outdoor mobility, transportation) in their daily lives while using their wheelchairs. | 10 items on a 6-point Likert scale ranging from 1 (completely disagree) to 6 (completely agree) (score = 0, does not apply). Total score ranges from 10 to 60 (higher score corresponds to higher perceived functional independence) | mobility device users (10; Giesbrecht et al., 2009) |
|  | FMA | 12 | Perceived functional independence of all mobility AP users in performing 9 functional tasks (adapted by FEW) | 10 items on a 6-point Likert scale ranging from 1 (completely disagree) to 6 (completely agree) (score = 0, does not apply). Total score ranges from 10 to 60 (higher score corresponds to higher perceived functional independence) | wheelchair recipients (344; Toro et al., 2016), mobility ADs users (41 Kumar et al., 2013) |
|  | WUSPI | 12 | Shoulder and wrist pain in wheelchair users during transfers, personal care, wheelchair mobility and activities of daily living | 15 items (self-rated on a 10 cm visual analog scale (VAS)); Likert scale 10-point: 1 (no pain) to 10 (worst pain ever experienced) | MWC users with SCI (24; Vincent et al., 2019), |
|  | CETI-m | 22 | changes in functional communication - self-perceived limitation of a person when communicating | 10 questions related to communication in different contextual situations, rated on a visual Likert scale (10-point scale). The total score is given by the sum of the scores (100-point scale). | ALS patients and caregivers (44; Londral et al., 2015) |
|  | GSRT | 22 | silent reading comprehension | 13 separate, paragraph-length stories followed by five multiple-choice comprehension (literal, inferential, critical, and affective) questions. Test yields raw scores, grade equivalents, age equivalents, percentiles, and a Silent Reading Quotient. | CP AAC AT users (7; Lund et al., 2006) |
|  | PPVT-R | 22 | comprehension of single word vocabulary | The examiner presented a word orally (175 stimuli of increasing difficulty), and the participants were asked to select the drawing (from a field of four) that best represented the meaning of the word. The raw score was converted to a standard score (mean standard score is 100±15) | CP AAC AT users (7; Lund et al., 2006) |
|  | TACL-R | 22 | comprehension of semantics, morphology and syntax | The examiner read a sentence aloud (120 items) and the participant selected the drawing (from three choices) that best represented the meaning of the sentence. | CP AAC AT users (7; Lund et al., 2006) |
|  | COGNISTAT | 22 | Cognitive impairments in seven major cognitive domains: orientation, attention, language, spatial skills, memory, calculation, and reasoning. The examiner also rates the level of consciousness | Performance is rated as average (no impairment), mildly impaired, moderately impaired, or severely impaired. There is initial item as a general screen for each domain, excluding orientation and memory. If the screening item is passed, the underlying ability is considered normal and a maximum score is assigned. If the patient fails the screening item, additional items are administered to establish performance level. Total score does not existed, only a domain-specific representation of the patient’s cognitive status. | older adults with or without dementia (27; Nishiura el al., 2013) |
|  | DBD-13 | 22 | Frequency of behavioral and psychological symptoms | 13 items on a 4-point Likert-type scale ranging from 0 (never) to 4 (all the time). Total score ranges from 0 to 52, with higher scores indicating severe impairment. | older adults with or without dementia (27; Nishiura el al., 2013) |
|  | MMSE | 22 | Global cognitive function - orientation, concentration, attention, verbal memory, naming and visuospatial skills. | Total score ranges from 0 to 30, with lower scores indicating more sever impairment. | older adults with or without dementia (27; Nishiura el al., 2013) |
| *Participation* | LLDI | 12 | Frequency and perceived limitation of participation in 16 life tasks (social role domain e.g., visiting friends and family in their homes, taking part in active recreation and personal role domain e.g., taking care of own health; taking care of own personal care need) | The disability component (16 items) has two types of score for each item, on a 5-step likert scale: "How often do you…?" (frequency) from 1 (never) to 5 (Very often); "To what extent do you feel limited in…?" (limitation) from 1 (Completely) to 5 (Not at all). Higher score corresponds to less disabled | Multiple sclerosis and spinal cord injury/disease (128; Mortenson et al., 2015) |
|  | NOMO 1.0 | 12 | Effectiveness of mobility devices in assessing mobility-related participation - frequency of mobility-related participation and ease/difficulty in mobility when performing activity (e.g., do cleaning, go to restaurant, shop for groceries, work, visit family or friend) | the scale is composed of 24 items, but only 20 items belong to the mobility-related participation domain. the frequency dimension ranges on an 8-point ordinal scale from “daily” to “never,” whereas the ease/difficulty dimension is rated on a 5-point ordinal scale ranging from “very easy” to “very difficult" ("I don't know" response in avaiable). The NOMO 1.0 does not provide a total score | powered mobility device recipients (180; Sund et al., 2015) |
|  | CHART | 12 | level of handicap in a community setting - 6 domains: physical independence, cognitive independence, mobility, occupation, social integration, and economic self-sufficiency | 32 items with different types of scores for each question. For each CHART dimension, a scoring procedure allows a score from 0 to 100 points: Higher scores indicate less handicap/greater degree of social and community participation. The short version of CHART is composed by 19 questions and evaluates the same domains. | wheelchair users with tetraplegia caused by SCI (30; Hastings et al., 2011), wheelchair recipients (344; Toro et al., 2016), mobility ADs users (41; Kumar et al., 2013) |
|  | ATOP/M | 12 | Impact of mobility devices on the level of activity and participation of the user with and without assistive technology: self-perceived difficulties that mobility AP users encounter performing many activities and roles. | 68 items on a 5-point Likert scale from 1 (Unable to do) to 5 (without any difficulty). Computer-assisted testing was used in the administration of this measure, and a T score ranging from 0 to 100 for each sub-domain (activities and participation) was calculated. | Multiple sclerosis and spinal cord injury/disease (128; Mortenson et al., 2015), power mobility users (115; Mortenson et el., 2014) |
| *Caregiver Burden* | CATOM | 12-15 | Impact of AT interventions on the burden experienced by informal caregivers | 18 items on a 5-point Likert Scale from 1 (Never) to 5 (Nearly always). It is administered via a semistructured interview and is divided into three parts: 1) caregivers identify activities in which they help; 2) they use a Likert scale to rate the burden they experience, with 14 aspects (e.g., physical demands, worry, time demands) of a selected activity; 3) caregivers rate how frequently they experience an overall sense of burden considering all activities in which they help. All scores were calculated by adding item scores: higher cumulative scores indicate greater feelings of burden. | PWC users' caregivers (35; Rushton et al., 2017+35; Labbè et al., 2019), AT users' caregivers (88; Mortenson et al., 2013), AT users’ caregivers (44; Mortenson et al., 2015), AT mobility interventions recipient and caregivers (110; Mortenson et al., 2018) |
|  | ZBI | 15 | Caregivers' burden - how the patient's disability impacts the quality of life, psychological suffering, guilt, financial difficulties, shame and the caregiver's social and family difficulties. | 22 items on 5-point Likert scale 0 (never) to 4 (nearly always). Total score is calculated by summing all item scores and range between 0 (null care load) and 88 (maximum care load level). A score higher than 24-26 identifies caregivers needed investigation and any support intervention | dementia (495; Gathercole et al., 2021) |

Multimedia Appendix 2. Information (evaluated domain, AT category, description, scoring) on available and used instruments to provide an AT outcome measure assessment. Colon Population (number of participants in the study; citation of the study).
