# Supplementary material for: Available Assistive Technology Outcome Measures: Systematic Review
Source: JMIR Rehabil Assist Technol. 2023 Nov 15;10:e51124. doi: 10.2196/51124 (PMC10687703; doi:10.2196/51124)
Supplement: Multimedia Appendix 3 [file rehab_v10i1e51124_app3.docx]

| AT Category | Satisfaction | Usability | Confidence | Psychosocial Impact | Quality of Life | Functional Efficacy | Participation | Caregiver Burden |
| --- | --- | --- | --- | --- | --- | --- | --- | --- |
| 12 | x | x | x | x | x | x | x | x |
| 15 | x | x |  | x | x | x |  | x |
| 22 | x |  |  | x | x | x | x |  |
| 24 | x | x |  | x |  | x |  |  |

Multimedia Appendix 3: Evaluable Domains for each Categories of AT with the available tools
